# Can data from paediatric cohorts solve the COVID-19 puzzle?

**DOI:** 10.1371/journal.ppat.1008798

**Published:** 2020-09-09

**Authors:** Lien Anh Ha Do, Jeremy Anderson, Edward Kim Mulholland, Paul V. Licciardi

**Affiliations:** 1 Infection and Immunity, Murdoch Children’s Research Institute, Melbourne, Australia; 2 Department of Paediatrics, The University of Melbourne, Melbourne, Australia; 3 Epidemiology and Population Health, London School of Hygiene and Tropical Medicine, London, United Kingdom; University of Pittsburgh, UNITED STATES

## Abstract

COVID-19, caused by SARS-CoV-2, is significantly more severe in adults than in children. The biological reasons for this difference remain to be elucidated. We have compared the most recent virological and immunological data related to COVID-19 between adults and children and contrasted this with earlier data from severe acute respiratory syndrome (SARS) caused by the related SARS-CoV-1 in 2003. Based on these available data, a number of hypotheses are proposed to explain the difference in COVID-19 clinical outcomes between adults and children. NF-kB may be a key factor that could explain the severe clinical manifestations of COVID-19 in adults as well as rare complications associated with paediatric inflammatory multisystem syndrome temporally associated with SARS-CoV-2 (PIMS-TS) in paediatric COVID-19 patients.

COVID-19 is a once-in-a-century pandemic caused by the completely new emerging pathogen SARS-CoV-2, which crossed the species barrier and first spread to the human population in December 2019 [1]. At the time of writing, global epidemiological data on COVID-19 indicate less than 2% of infections have occurred in children. Of these, most are mild, with 12.9%–21% asymptomatic and <0.2% severe (8 reported deaths), in contrast to older adults, in whom 20% of cases require hospitalization and 5% are critical, with deaths occurring in 70% of critical cases [2–12]. Prevalence of asymptomatic SARS-CoV-2 infection in adults is reported to be around 43%–87.9% of laboratory confirmed cases from systematic screening in longitudinal studies [13–15]. No such data exists in children. Cross-sectional systematic screening of SARS-CoV-2 in general populations in European COVID-19 hotspots showed a low detection (or even no detection) of SARS-CoV-2 in paediatric populations [16, 17]. In a study of 391 adult COVID-19 cases and 1,286 close contacts in Shenzhen, China—one of the first hotspots of the pandemic—it was found that children appear to be as susceptible to infection as adults but are less likely to develop severe disease [18]. The reasons why children are either less susceptible to and/or have less severe illness than adults should be a prioritized research question. To facilitate this, we provide some hypotheses on the susceptibility of SARS-CoV-2 in children using the most recent COVID-19 data in adults and children and knowledge gained during the SARS epidemic in 2003, which was caused by the related SARS-CoV-1 [19]. Given the high degree of relatedness between SARS-CoV-2 and SARS-CoV-1 (79% genome homology) [20], valuable insights can be gleaned to offer promising avenues of investigation.

## Differences in ACE2 expression between adults and children

The angiotensin converting enzyme II (ACE2) is the host receptor for both SARS-CoV-1 and SARS-CoV-2 [21]. The receptor-binding domain (RBD) of the S (spike) protein of SARS-CoV-2 has 10–20 times higher affinity to ACE2 than SARS-CoV-1 [22]. This may explain the increased infection rate and transmissibility of SARS-CoV-2.

In humans, ACE2 can be found on several cell types, such as type II alveolar cells, epithelial cells of respiratory and digestive tracts, cholangiocytes, proximal tubule cells of the kidney, bladder urothelial cells, myocardial cells, endothelial cells, macrophages, and T cells [23, 24]. A recent report found that nasal epithelium including goblet cells, ciliated cells, and cornea also express ACE2. Among these, the nasal epithelium has the highest ACE2 expression [25]. Limited data exists on differences in ACE2 expression between children and adults. In the lung, conflicting data on ACE2 expression by age has been reported [26, 27], while recent data showed significantly lower expression of ACE2 in the nasal epithelium of young children (4–9 years old) compared with older children (10–17 years old), young adults (18–24 years old), and older adults (≥24 years old) [28]. This may be one explanation for the lower rates of SARS-CoV-2 infections in children, but more studies are needed. Moreover, viral detection in different tissues and body fluids, mainly respiratory and intestinal samples, have been increasingly reported in hospitalized COVID-19 cases in adults [29] and also children, although in a limited number of cases [30, 31]. Differential expression of ACE2 and/or coreceptors on certain cell types may be sufficient to lead to variations in disease severity by age [32].

Moreover, the expression of ACE2 in airway epithelial cells was demonstrated to be a dynamic process related to cellular differentiation states [33]. ACE2 expression on airway epithelial cells was reported to be up-regulated by type I and II interferons, a tissue-host response against virus, which paradoxically also increased SARS-CoV-2 binding [34]. Increased ACE2 expression in influenza, tuberculosis, and HIV coinfections were also observed when compared to matched controls in an in vitro airway epithelial cell model of SARS-CoV-2 infections [34]. SARS-CoV-2 therefore appears to have a unique capacity to exploit the host interferon response to directly facilitate viral entry. Further research is required to understand whether other viral coinfections, especially those which are more frequently observed in children than in adults, also induce a strong interferon response and may impact both the susceptibility to and dynamics of host-SARS-CoV-2 infection [34].

Risk factors such as older age (>60 years), as well as comorbidities such as cardiovascular diseases, diabetes, and hypertension, are associated with the development of acute respiratory distress syndrome (ARDS) and death in COVID-19 [35]. ACE2 plays a key role in the Renin-Angiotensin-Aldosterone System (RAAS) to regulate vasoconstriction and sodium retention, while also being the host receptor for SARS-CoV-2. It is likely that activation of ACE2 is critical for the development of ARDS through RAAS [36]. Patients with hypertension and diabetes are often prescribed RAAS inhibitors (e.g., ACE inhibitor and angiotensin II receptor blockers [ARBs]), which may affect ACE2 expression, promoting COVID-19 disease severity. A study analyzed the expression of ACE2 and two host cell proteases (TMPRSS2 and ADAM17, as co-host factors for virus entry) in 1,051 lung tissue samples and correlated this with medication use. The use of ACE inhibitors was associated with a significantly lower expression of ACE2 and TMPRSS2, while the use of ARB did not alter expression of these genes [37]. These recent data suggest no harmful effect of RAAS inhibitors on COVID-19 severity, despite hypertension being a dominant risk factor for severe COVID-19 [38]. The factors driving an increased risk of severe COVID-19 in these comorbidity populations remains an important question for the field.

In summary, research investigating high ACE2 expression as a potential susceptibility mechanism and therapeutic target for severe COVID-19 is still ongoing [39–41]. The potential role for a coreceptor in viral entry requires further investigation and may be an important factor in the susceptibility to severe COVID-19 disease.

## Differences in immune responses between adults and children

A large gap in our knowledge still exists in explaining the difference in susceptibility between adults and children to severe COVID-19 disease. The role of the immune system is suggested to play a key role. We propose two hypotheses to explain the different outcomes between adults and children with COVID-19: 1) pre-existing immune responses in children and 2) an overreactive immune response in adults.

### Pre-existing immune responses in children

Children, especially during their first three years of life, are frequently exposed to a range of human coronaviruses (hCoV-229E, -HKU1, -NL63 and -OC43) [42]. A 2010–2015 study in Guangzhou, one of the hotspots for COVID-19, addressed the prevalence of these hCoV infections, particularly OC43, which is a betacoronavirus, the same group of coronavirus as SARS-CoV-1 and SARS-CoV-2. OC43 was mostly seen in children less than 3 years of age (20%), less frequently in children under 15 years of age (0.5% to 20%) and relatively uncommon in adults older than 35 years (<0.2%) [43]. Some studies have shown that neutralizing antibodies (NAbs) were detected in only 33%–46% of hCoV infections and were higher in older adults (aged ≥60 years) compared to young adults [42]. Moreover, reinfection in children and adults with hCoV is also common, suggesting a lack of long-term antibody-mediated protection [42]. Importantly, these NAbs generated from hCoV infections were not cross-reactive with SARS-CoV-2 or SARS-CoV-1 [44]. In contrast, antibodies specific for SARS-CoV-1 were shown to be cross-reactive with SARS-CoV-2 [45], but NAbs and memory B cell responses were short lived [46]. Together it is unlikely that pre-existing NAbs induced by other hCoV during early childhood explain the low rate of severe SARS-CoV-2 infections in children.

The role of nonspecific effects of childhood vaccines has also been suggested to provide some protection against COVID-19, although currently there is no data describing such effects. A previous study found no cross-reactive Abs against SARS-CoV-1 in a mouse model and the T-cell response induced following receipt of all childhood vaccines did not prevent SARS-CoV-1 infection in Vero cells [47]. The BCG vaccine is perhaps the most widely recognized vaccine for inducing heterologous protection against non-tuberculosis (TB) infectious diseases, mainly due to its reported effects on trained immunity [48, 49]. In a challenge model using live attenuated yellow fever virus vaccine in healthy adults, trained immunity due to BCG vaccination provided a rapid local antimicrobial response through interferon and other cytokine production by monocytes/macrophages that eliminated the virus [48]. From epidemiological data, in countries without universal BCG vaccination policies that have a high number of confirmed COVID-19 cases and deaths (e.g., Italy, the Netherlands, and the United States of America), the paediatric COVID-19 pattern is similar to China, where BCG is given at birth in the national immunization program [2–9]. This suggests that it is unlikely that BCG given during childhood would have an effect on COVID-19 susceptibility or severity later in life. These data highlight the need for further research across age groups, with a focus on immune responses to SARS-CoV-2 in recent recipients of BCG vaccines. Of note, SARS-CoV-1 has the ability to delay early activation of interferon responses, especially interferon ß, to facilitate viral replication, leading to an intense interferon response inducing cytokine storm [50, 51]. Timing when using type 1 interferon has been shown to be critical in clinical trials of SARS-CoV-1 treatment. Type 1 Interferon that was introduced shortly after infection had positive effects. However, when introduced later, it failed to reduce viral load, leading to more side effects [52]. It is still unknown if SARS-CoV-2 could also have this capacity to manipulate interferon responses. If the delayed interferon response is true for SARS-CoV-2 infections, this would significantly challenge the potential impact of BCG vaccination in preventing SARS-CoV-2 infections or reducing COVID-19 severity, particularly if protection is only through trained immunity, not by heterologous T-cell responses. The ongoing randomized controlled trial of BCG vaccine in health care workers across the globe that first commenced at the Murdoch Children’s Research Institute, Australia (BRACE study [53]), will provide evidence as to whether BCG vaccine can prevent severe SARS-CoV-2 infections. Until results of these trials are available, the use of BCG vaccine for prevention of COVID-19 is not recommended, in line with WHO recommendations [54].

### Over-reactive immune responses in adults

Increasing clinical and preliminary antibody data from COVID-19 patients have supported the idea of an “over-reactive” immune response in adults compared to children. These over-reactive immune responses were observed through a stronger antibody response and a more intense proinflammatory response characterized by cytokine storm in adults compared with children.

There is conflicting data on the correlation between antibody levels and clinical severity in adults with COVID-19 compared to what was observed during SARS. In COVID-19, those with severe disease also developed a peak antibody response more rapidly, as was observed in critical cases of SARS [55, 56]. Interestingly, a recent study among mild COVID-19 cases found elderly (60–85 years) and middle-aged (40–59 years) patients were more likely to induce higher titers of NAbs than younger patients (15–39 years) [57]. In SARS, adults who developed antibodies within two weeks of symptom onset were likely to be >60 years of age, experience a shorter survival time, and have a higher mortality rate [58], while NAbs were shown to correlate with viral load and disease severity [59–61]. In a case report of one mild COVID-19 case, the detection of IgG appeared before the reduction of viral load and improved clinical presentation [61]. A recent longitudinal data in hospitalized COVID-19 cases showed increased SARS-CoV-2 NAb titers correlated significantly with reduced viral load and infectious shedding [62]. However, in a series of mild cases (but without viral load data), the titer of NAbs positively correlated with C-reactive protein (CRP) in blood—an inflammatory marker—and negatively correlated with lymphocyte counts [57], which was more apparent in older patients at admission [57].

“Cytokine storms” have been observed frequently in adults with COVID-19 or SARS, presenting as ARDS. No such common features have been observed in severe cases in children [63]. Adults are usually less prone to ARDS than children in other viral infections; however, it is clear in COVID-19 that the prevalence of ARDS in adults is much higher [64]. IL-6 is the predominant cytokine observed in COVID-19 adult cases [36] and is significantly correlated with ICU admission [2]. Of note, given the role of IL-6 in cytokine storm, a monoclonal antibody targeting IL-6 receptor (tocilizumab) has been used in a number of trials due to promising results from studies in China and Italy [65, 66]. In COVID-19, viral load, IL-6, and severity are strongly correlated in adults but surprisingly not in children. In a cohort of 76 adult patients in China, a high viral load and a long virus-shedding period was associated with severity of disease [67]. In contrast, a six-month old boy in Singapore had a persistent and higher SARS-CoV-2 viral load than his parents, yet he remained asymptomatic [30]. In children with COVID-19, IL-6 levels are occasionally increased in severe cases [68], but in general, levels are within the normal range [31, 69–71]. One hypothesis is related to the viral N protein, where there is a “signature” sequence (amino acids from 86–96 and 341–422) only found in SARS-CoV-1 and SARS-CoV-2 but not in other human coronaviruses. In SARS disease, this N signature sequence was shown to activate IL-6 expression by directly binding to NF-kB p65 transcription factor regulatory elements [72]. NF-kB is an important regulator of proinflammatory cytokine production and B-cell function, with expression reported to increase with age [73]. In individuals >65 years, the NF-kB transcription factor was highly expressed even in resting CD4+ T cells [73]. In mouse models of SARS, activation of NF-kB was associated with pathogenesis of disease, while SARS-CoV-1–vectored vaccines expressing N gene increased proinflammatory cytokines and chemokines, especially IL-6 [74–76]. Activation of NF-kB leading to ARDS in COVID-19 could be linked to the higher affinity of the S protein of SARS-CoV-2 with ACE2, leading to the release of angiotensin II via angiotensin receptor type 1, activating NF-kB [77, 78] and releasing proinflammatory cytokines such as IL-6 [78]. The findings to date on viral load, IL-6 level, and clinical presentations in children versus adults appear to align well with the potential role of NF-kB activation.

In severe COVID-19 cases, around 16%–49% of cases were associated with thrombotic and coagulation complications [79, 80]. NF-kB regulators are also involved in coagulation and thrombotic processes, specifically in platelets, endothelial cells, neutrophils, and NETs [81]. Reports suggest elevated levels of blood neutrophils and reduced platelets as early indicators of SARS-CoV-2 infection, predicting severe respiratory disease and severe outcomes in adults [82] but not in children [63, 83]. NETs were reported to promote arterial and venous thrombosis [81], and high levels of all three markers of NETs (cell-free DNA, MPO-DNA, and Cit-H3) were detected in the serum of COVID-19 patients, with cell-free DNA and MPO-DNA levels significantly higher in patients requiring mechanical ventilation versus inpatients breathing room air [82]. Importantly, several studies have now highlighted the importance of the NF-kB signaling pathway in COVID-19 progression [84]. This requires further investigation in larger studies to confirm these findings and provide a basis for the over-reactive immune responses observed in adults with COVID-19.

From March to early May 2020, warnings of a link between Kawasaki Disease (KD), a very rare cause of autoimmune systemic vasculitis in children and COVID-19 leading to severe illness in some children has raised serious questions about the manifestations of COVID-19 in children. Clinical observations in those children showed some overlap between atypical KD or KD (toxic) shock syndrome. Those patients represented a multisystem hyper-inflammatory state, termed “paediatric inflammatory multisystem syndrome temporally associated with SARS-CoV-2” (PIMS-TS). Patients with PIMS-TS were generally older than those with KD or KD shock syndrome and had higher levels of markers of cardiac injury [85–87]. One earlier case control study reported an association between KD and a newly discovered human coronavirus named New Haven coronavirus (hCoV-NH), most closely related to hCoV-229E but not from the same genera as SARS-CoV-1 and SARS-CoV-2 [88]. This finding is still debated [89], and the predisposition of the host, in addition to the antigenic properties of the virus, has been recognized as an important factor in the pathological features of KD. Intriguingly, the NF-kB signaling pathway was the top common pathway shared between KD and acute viral infections in children [90] and specifically NF-kB p65 was found to be excessively activated during the acute phase of KD, as a result of NF-kB p65 subunit directly interacting with the N protein of SARS-CoV-1 [91]. These data suggest that further research is needed to address whether NF-kB is a key disease-modifying target in children.

If a similar role for the N protein and NF-kB is demonstrated for COVID-19 as for SARS in adults, then it is critical that new antiviral treatments are given early during the onset of disease to block the proinflammatory signals leading to cytokine storm. However, early interferon antiviral treatment would be clinically challenging, since some COVID-19 patients were reported to have a high viral load at presentation before symptom onset [55]. Other treatment options targeting IL-6 by blocking its receptors or directly inhibiting IL-6 (e.g., tocilizumab, siltuximab, sarilumab) may be better suited in these cases.

## Conclusion

Given the unique position of children in this pandemic, a better understanding of the factors associated with the differential susceptibility between adults and children should be a high priority for research. This question can be integrated to a household contact case-control study aiming to provide a better understanding about disease transmission and inform correlates of protections and therefore preventive strategies. Using a multidisciplinary approach involving high-dimensional immunological, genomic, and proteomic techniques is likely to reveal crucial insights into viral and protective host factors. Such information is urgently needed as part of the overall strategy to solve the COVID-19 disease puzzle.

## Abbreviations

ACE2: angiotensin converting enzyme II
ADAM17: A disintegrin and metalloprotease domain 17
ARB: angiotensin II receptor blocker
ARDS: acute respiratory distress syndrome
BCG vaccine: Bacillus Calmette-Guerin vaccine
BRACE: BCG Vaccination to Protect Healthcare Workers Against COVID-19
Cit-H3: citrullinated histone 3
COVID-19: Coronavirus Disease of 2019
CRP: C-reactive protein
hCoV: human coronavirus
hCoV-NH: New Haven coronavirus
MPO-DNA: myeloperoxidase-DNA
IgG: immunoglobulin G
IL-6: interleukin 6
KD: Kawasaki Disease
Nab: neutralizing antibody
NET: neutrophil extracellular trap
NF-kB: nuclear factor kappa B
PIMS-TS: paediatric inflammatory multisystem syndrome temporally associated with SARS-CoV-2
RAAS: renin-angiotensin-aldosterone system
RBD: receptor-binding domain
S: spike
SARS: severe acute respiratory syndrome
TMPRSS2: Transmembrane Serine Protease 2
TB: tuberculosis
